# Epidemiology and diagnosis of feline panleukopenia virus in Egypt: Clinical and molecular diagnosis in cats

**DOI:** 10.14202/vetworld.2018.578-584

**Published:** 2018-05-04

**Authors:** Romane A. Awad, Wagdy K. B. Khalil, Ashraf G. Attallah

**Affiliations:** 1 Department of Parasitology and Animal Diseases, Veterinary Division, National Research Center, 33 Bohouth St., 12622 Dokki, Giza, Egypt; 2 Department of Cell Biology, National Research Center, 33 Bohouth St., 12622 Dokki, Giza, Egypt; 3 Department of Microbial Genetics, National Research Center, 33 Bohouth St., 12622 Dokki, Giza, Egypt

**Keywords:** cats, Egypt, ELISA, epidemiology, feline panleukopenia, feline panleukopenia virus, polymerase chain reaction, sequencing

## Abstract

**Aim:**

This work aimed to study epidemiology and diagnosis of feline panleukopenia virus (FPV) using clinical examination, direct ELISA, RNA viral isolation and identification, and knowing phylogenetic tree of our isolate.

**Materials and Methods:**

One hundred and sixty-five cats of different ages and sex were examined. Each cat was examined clinically to detect the clinical manifestations of the disease showing symptoms suggestive of feline panleukopenia (FP) as well as ELISA, and polymerase chain reaction (PCR) amplification analyses were conducted.

**Results:**

Our finding includes (a) clinical signs detected in 165 of 165 cats were in the form of lethargy, fever, anorexia, thirst, vomiting, diarrhea, dehydration, and leukopenia. (b) ELISA results revealed that 66 of all examined cats were positive for FPV. (c) The amplification products from all positive samples were confirmed as FPV (VP1) gene by nucleotide sequences analysis, in which 75 samples were positive using PCR amplification for the FPV. (d) Statistical evaluation of ELISA results in comparison to PCR findings. ELISA showed 88%, 100%, and 94.5% for sensitivity, specificity, and accuracy, respectively, while the prevalence of FP among the examined population was 45%. No effect of sex, breed, and age on ELISA results as recorded using Chi-square analysis.

**Conclusion:**

The results of the sequence analysis indicated that PCR products of the FPV cDNA exhibited very low variation in their nucleotide sequence of all isolates compared with the published FPV genome, which could be suggested that FPV appears to be genomically stasis compared with other Parvoviruses. The genome sequence of FPLV strain in this study has been deposited in GenBank under the accession number KY466003. Our isolate closely related 100% to isolates from Portugal, which might be the origin of infection to Egypt through importation of cats.

## Introduction

Feline panleukopenia (FP) is a highly contagious often fatal viral disease affecting domestic and wild felids. The disease is clinically manifested by severe depression, vomiting, dehydration, and enteritis diarrhea and is often fatal. A marked decrease in circulating white blood cells (WBC) has been recorded [1-3]. The virus is acquired through oronasal route and targets rapidly dividing cells, especially the epithelium crypt of the small intestine, lymphoid tissue, and bone marrow [1-3].

Moreover, feline panleukopenia virus (FPV) is closely related to, especially, the canine parvovirus-2 (CPV-2) to CPV-2 and its antigenic variants, designated CPV-2a, CPV-2b, and CPV-2c. Several studies suggested that FPV is the ancestral origin of CPV-2 [4,5]. Despite their relatedness, antigenic differences between FPV and CPV-2 exist and distinguishable using monoclonal antibodies [6,7]. Although the genetic and amino acid differences among these viruses are small, they occur differently in the important antigens of VP2, the major capsid protein of the viruses [7].

Treatment of the disease should be aimed at controlling the secondary bacterial infection, combating dehydration, and restoring electrolyte balance [8]. The commercially available vaccines for FPV virus include the modified live virus or an inactivated virus vaccine [1]. Cat not vaccinated or not received the booster dose of FPV vaccines is at risk [9]. To our knowledge, no published data are available regarding the FPV infection in Egypt. Therefore, the fast diagnosis and detection of the virus are important in countries in which the disease may spread quickly such as in Egypt. Detection of FPV infection is important not only for diagnostic purposes but also to control infection in the populations such as those at low facilities against the virus treatment.

As different regular tools such as clinical examination and direct ELISA for the qualitative detection of FPV are widely used with many diagnostic disadvantages; the current study was aimed to overcome this inaccurate detection using molecular assays to identify the FPV from different resources such as blood and feces from live infected cats.

## Materials and Methods

### Ethical approval and informed consent

We informed and received the permission of the owners of cats included in this study for taking samples used in this work. Samples were collected as per standard sample collection procedure without any harm to animals.

### Chemicals

For molecular analysis, Trizol was bought from Invitrogen (Carlsbad, CA, USA). The reverse transcription and polymerase chain reaction (PCR) kits were obtained from Fermentas (Glen Burnie, MD, USA). For ELISA analysis, direct ELISA kits were purchased from Sigma (St. Louis, MO, USA).

### Experimental animals

One hundred and sixty-five diseased cats from different ages and sex were examined. Each cat was examined clinically to detect the clinical manifestations of the disease.

### Sampling

Fecal samples were collected from all clinically infected cases and checked by rapid ELISA test (rapid FPV Ag test kit for qualitative detection of viral antigen in feces of all examined cases) and virus isolation [1,9]. For isolation of FP virus blood samples were collected from infected cats [10]. The cats presented clinical signs of Panleukopenia including vomiting, diarrhea, depression and anorexia were used for virus isolation. Collected samples were stored in 2 ml microtubes at a temperature of -20°C.

### Clinical examination

One hundred and sixty-five cats were received at a clinic, namely, German Veterinary Clinic at October 6 located in Giza Governorate, Egypt. Cats subjected to general and specific clinical examination according to Gaskell *et al*. [1] were examined by measuring body temperature, examination of buccal and conjunctival mucous membranes, examination of superficial lymph nodes by palpation and abdominal palpation. Also, appetite, body condition, skin, respiratory illness, digestive disturbances e.g., vomiting and diarrhea were recorded for each infected case. The severity of the clinical signs observed in this study was recorded as mild, moderate, and severe. History of the examined cats including breed, sex, age, past medical data history, and registered vaccination [11] were taken into consideration to identify any correlation between the infection and one or more of these parameters.

### ELISA

Direct ELISA (the antigen rapid FPV Ag test kit, Bionote Inc., Korea) for the qualitative detection of FP viral antigen in feline feces was carried out on 165 fecal samples of cats showing clinical signs of FP viral infection [9].

WBC count was carried out on whole blood collected from cats in laboratory tubes containing EDTA. These samples of infected cats were positive for rapid antigen FPV test kit and showed clinical signs and dehydration estimated at 5-7% as recommended by Duncan *et al*. [10] and Smith [12].

### RNA extraction

RNA was extracted from blood samples of infected cats. Blood samples were collected in 1 ml EDTA as an anticoagulated solution. Total RNA was extracted from whole blood samples within 60 min of collection using the QIAamp Blood Mini Kit (Qiagen, Hombrechtikon, Switzerland). Approximately 200 µL of the blood samples from each cat were homogenized and mixed with the lysis buffer in the autoclaved extractive tube. Afterward, total RNA was dissolved and preserved in diethyl pyrocarbonate-treated water up to use. To assess the RNA yield and purity of the total RNA, RNAse-free DNAse I (Invitrogen, Germany) was used to digest DNA contamination. A small drop of isolated RNA was examined photospectrometrically at 260 nm. The purity of total RNA was determined between 1.8 and 2.1 to be good purified when it was examined by photospectrometer at the 260/280 nm ratio. To avoid RNA damaging, aliquots of RNA were prepared after isolation for either reverse transcription reaction or otherwise for storing at −80°C up to use and stored at −80^o^C until further use [13].

### Primer design

The specific primer used in this study was designed using Primer3 software (version 0.4.0, http://primer3.ut.ee) according to complete genome of PLV genome as illustrated in Table-1.

**Table-1:** Primers used in cDNA amplification.

Primer name	Oligonucleotide sequence (5′-3′)	Estimated product size^a^
Left primer	TGC CTC AAT CTG AAG GAG CT	881-1105 bp
Right primer	TTT CAT CTG TTT GCG CTC CC	

a Based on available FPV genome sequences. FPV=Feline panleukopenia virus

### First-strand cDNA synthesis and PCR amplification

Extracted total RNA samples were reverse transcribed into cDNA. First-strand cDNA was synthesized in duplicate using the High Capacity cDNA Reverse Transcription Kit (Applied Biosystems, Rotkreuz, Switzerland) and oligo (dT) primer according to the manufacturer’s instructions.

### Statistical analysis

Statistical analysis was done to study the effect of sex, breed, and age on the results of ELISA using Chi-square [16].

## Results

### Clinical examination

**Figure-1:**
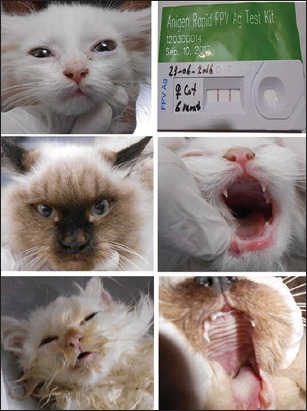
Clinical pictures of feline panleukopenia symptoms on cats.

The severity of the disease varied from mild transient fever and leukopenia to severe, peracute form that was fatal usually in kittens. Clinical signs are summarized in Table-2. Some of the clinical signs of the disease encountered in infected cats in this study are shown in Figure-1.

**Table-2:** The clinical signs detected in examined cats.

No. of cases	Breed		Age (months)	Sex		Lethargy	Fever	Anorexia	Thirst	Vomiting	Diarrhea	Dehydration	Leukopenia
	
	Persian	Siam		Male	Female								
20	20	-	1	10	10	Severe	Severe	Severe	Severe	Mild	Severe	Over 12%	Mild
30	25	5	2	18	12	Severe	Severe	Severe	Severe	Mild	Severe	10-12%	Mild
45	30	15	3-4	20	25	Severe	Severe	Severe	Severe	Severe	Severe	8%	Mild
25	22	3	6-7	12	13	Severe	Severe	Severe	Severe	Severe	Moderate	6-8%	Moderate
20	20	-	8-10	12	8	Moderate	Severe	Severe	Severe	Severe	Moderate	6-7%	Moderate
15	15	-	12	6	9	Mild	Moderate	Severe	Mild	Severe	Moderate	5%	Moderate
10	10	-	24	5	5	Mild	Mild	Severe	Mild	Severe	Moderate	5%	Moderate

Table-3 shows dehydration rate and WBC counts in infected cats. The results showed that dehydration rates were ranged from 5% to 7%. Normal WBC were ranged from 5.5 to 15.4×10^9^/L.

**Table-3:** Dehydration rate and WBC counts for examined cats.

Cats	Dehydration rate	Normal WBC count	Counted values
20	6-7%	5.5-15.4×10^9^/L	1.8×10^9^/L
15	5%	5.5-15.4×10^9^/L	1.9×10^9^/L
10	5%	5.5-15.4×10^9^/L	1.7×10^9^/L

WBC=White blood cell

### ELISA result for FPV

Table-4 shows the positive and negative cats examined for ELISA FPV. Furthermore, infected cats were more observed in young cats (1-7 months) than older cats (Table-5). The results showed that a number of infected male cats with FPV was relatively higher than those of FPV-infected female cats. The percentage of infected male cats was 39.5% of all examined males, while infected female cats exhibited 40.5% of all examined females.

**Table-4:** Positive and negative male and females cats examined for FPV ELISA.

Total	n (%)	

	Male	Female
Positive	34 (39.54)	32 (40.50)
Negative	52 (60.46)	47 (59.50)
Total	86	79

FPV=Feline panleukopenia virus

**Table-5:** Distribution of ELISA results between different age groups.

Age group	Age (months)			Total

	1-7	8-12	Over 12	
Positive	45	17	4	66
Negative	120	35	10	165

### Molecular determination for FPV

**Figure-2:**
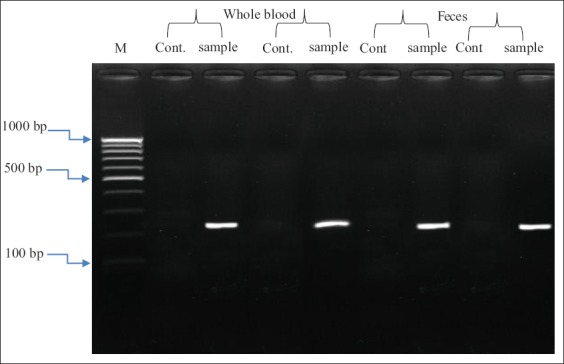
Amplified cDNA of normal (cont.) and infected (sample) feline panleukopenia virus (FPV) cat samples. Only positive FPV DNA bands were showed at 225 bp for the infected cats. M represents DNA markers.

To determine the FPV based on the molecular basis, the templates of cDNA collected from cat samples (blood and feces) with signs compatible with FP were used to detect the FPV gene in these samples. The cDNA of all samples was amplified in the conventional PCR assay using a primer that was previously designed according to complete CDS of capsid protein gene of FPV genome. The results revealed that the cDNA from all examined FPV samples exhibited clear bands of amplification with its primer with a 225 bp product size (Figure-2). The molecular size of the resulted PCR products corresponded to the expected size and no additional or non-specific bands were observed (Figure-2). In addition, healthy cats (control) exhibited negative FPV bands.

Analysis of the genomic region encompassed by this primer and its predicted amino acid sequence allowed discrimination of FPV from all its variants tissues. The amplification products from all positive samples were confirmed as FPV (VP2) gene by nucleotide sequences analysis. Analysis of the sequences of all products illustrated in phylogenetic tree showed a high degree of nucleotide homology. The sequence obtained for each isolate was aligned with the sequences available in the database using BLAST, and the sequences with the highest coverage and highest degree of similarity were selected.

**Figure-3:**
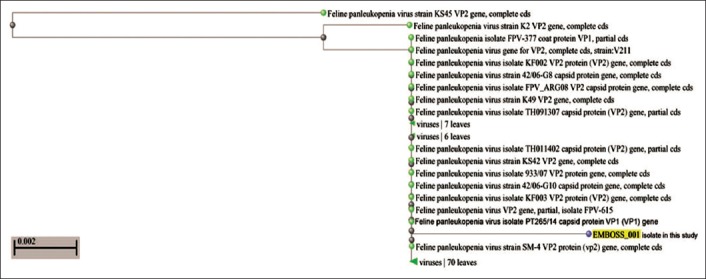
Phylogenetic tree constructed from the VP2 gene nucleotide sequences of the feline panleukopenia virus strain generated in this study and other sequences obtained from the GenBank database.

The identification of the FPV sequence in the current study was determined according to BLASTN programs version 2.5.1+ at the GenBank NCBI on the website http://blast.ncbi.nlm.nih.gov/Blast.cgi, using FASTA format. The assembled sequences were assigned by GenBank accession number sequences and revealed that all the examined sequences significantly matched, PT265/14 capsid protein (VP1) gene, with a maximum of 99% identity with the VP1 gene sequences of the strains available in the GenBank. Phylogenetic analyses of isolate with other sequences are depicted in Figure-3. The data in Figure-3 using phylogenetic tree analysis and the neighbor-joining method showed that three different clusters were formed, the first one contains only one strain of FPV KS45 VP2 gene, and the other remaining FPV strains found in the second cluster were divided into two subclusters. The FPV isolate in this study shows more similarity and closely related to strain PT265/14 than other isolates.

The nucleotide sequence was submitted using BankIt https://submit.ncbi.nlm.nih.Gov/subs/GenBank. The FPV gene fragment sequences were deposited in NCBI Nucleotide Database GenBank under accession no KY466003.

To compare between ELISA and molecular results for FPV identification, Table-6 reveals that ELISA results exhibited failure in some samples in FPV detection. However, the PCR assay revealed zero failure in detection of FPV compared to ELISA results. Moreover, the positive samples detected by PCR were more than the positive samples detected by ELISA. These results exhibited that the molecular analysis was more accurate compared to ELISA analysis.

**Table-6:** Comparison between ELISA according to PCR findings of examined cases of FPV.

Method of identification	ELISA		Total

	Positive	Negative	
PCR			
Positive	66(T+)	9(F−)	75
Negative	0(F+)	90(T−)	90
Total	66	99	165n

(T+): True positive, (T−): True negative, (F+): False positive, (F−): False negative and (n): Total number, PCR=Polymerase chain reaction

## Discussion

In the current study, we have used different methods for FP diagnosis such as clinical signs, direct ELISA for qualitative detection of FP viral antigen, and viral cDNA amplification to identify the FPV from cats’ blood and faces. According to our findings in this study, FP is a highly serious infectious disease of high percentage case affect fertility. The clinical course of FP varied from peracute to acute severe or mild form according to the development of clinical signs. The form of the disease is more severe in cats of age 3-10 months (acute form), while recorded clinical signs decreased in kitten 1-2 months old (peracute form) as recorded by Gaskel *et al*. [1]. Duncan *et al*. [10] reported that WBC count should be recorded in infected cats because the high WBC count helps to verify that these cats indeed have a virus infection. Therefore, the WBC count was achieved in the cats of the present study that have vomiting, diarrhea (5-7%), depression and anorexia as expected clinical signs of FP.

FP disease is closely related to CPV [1-3]. Moreover, detection of FPV by ELISA tool seems to be correlated with CPV-2 and its antigenic variants designated CPV-2a, CPV-2b, and CPV-2c. Hence, to avoid the overlapping between FPV and other related viruses, we have used different methods for FPV identification such as direct ELISA for qualitative detection and molecular determination including isolation of viral RNA, cDNA amplification, and sequencing. ELISA for the qualitative detection of FP antigen in feces is considered a most accurate test of high sensitivity and specificity as recommended by Abd-Eldaim *et al*. [17] and Tizzard [18], so we used in our study as field screening test for diagnosis of FP.

In our study, ELISA results recorded were 39.5% and 40.5% for positive male and female, respectively. Statistical analysis using Chi-square to detect the effect of sex, breed, and age in ELISA results revealed that no significant difference was detected between male and female, Siam and Persian breeds, and 1-7 months, 8-12 months, and over 12 months age groups.

Despite ELISA recorded as highly sensitive tool, detection of FP antigen by this test in feces of infected cats in some cases was not effective to identify the FP virus. Cats can shed FP viral antigen in faces after vaccination with live attenuated vaccine [17,18], and also, ELISA kit of FP viral antigen detection can detect antigens of other closely related parvoviruses. Hence, we used isolation of viral RNA, cDNA amplification, and sequencing as most accurate, specific techniques to avoid overlapping between FP and other related viruses in diagnosis.

FP viral isolation and PCR are gold standard test of this study to which ELISA test compared and evaluated [17, 19-21]. Results of PCR showed 44% and 37% for positive cats among examined male and female cats, respectively. The number of positive cases of PCR is 75 while 66 for ELISA. Hence, ELISA showed 88%, 100%, and 94.5% for sensitivity, specificity, and accuracy, respectively, while the prevalence of FP among all examined cats was 45% [12,20].

Furthermore, our study showed that PCR results of the cDNA amplification exhibited more number of positive results for FPV than those detected by ELISA results. These results were in agreement with those of Abd-Eldaim *et al*. [17], who found that most of the negative cases for ELISA FPV were positive when examined by PCR. These findings could be resulted from the high sensitivity of the PCR compared with ELISA and other detection methods.On the other hand, several studies revealed that some samples were positive by ELISA and negative by PCR methods [13-15,17]. These results could be interpreted that the cats were vaccinated live attenuated vaccine several days before collection of the samples [18]. Moreover, the negative results of the PCR could also be attributed to the presence of inhibitory compounds in the samples, nucleic acids degradation, or false-positive results of the ELISA.

The results of the sequence analysis in the present study indicated that PCR products of the FPV cDNA exhibited very low variation in their nucleotide sequence of all isolates compared with the published FPV genome in the GenBank. These results are similar to previous other studies suggesting that FPV appears to be genomic stasis compared with other viruses such as Parvoviruses [16,22].

The results of the present study exhibited that the percentages of infected male and female cats were lower by ELISA than by cDNA amplification. The percentages of male cats were positive by 39.5% for ELISA FPV and 77.9% for cDNA amplification, while infected female cats exhibited 40.5% for ELISA FPV and 78.5% for cDNA amplification of all examined females. Our findings were in great disagreement with those by Abd-Eldaim *et al*. [17], who found that most of the examined cats were strongly positive on ELISA. They suggested that most of the examined cats were vaccinated in which the positive results for ELISA could be related to vaccine of the FPV. However, most of the cats in the present study were not vaccinated, and the positive results on ELISA for FPV were more likely from field strains. Moreover, several studies for detection of CPV-2 and FPV in cats indicated that positive results of ELISA for these viruses could be attributed to modified-live vaccines [13-15].

## Conclusion

The present work was aimed to study epidemiology and diagnosis of Feline Panleukopenia Virus **(**FPV) in cats. The results showed that most of the negative examined cases for ELISA FPV were positive when examined by PCR The results of the sequence analysis indicated that PCR products of the FPV cDNA exhibited very low variation in their nucleotide sequence of all isolates compared with the published FPV genome, which could be suggested that FPV appears to be genomically stasis compared with other Parvoviruses. Our isolate closely related 100% to isolates from Portugal, which might be the origin of infection to Egypt via importation of cats.

## Authors’ Contributions

All authors participated equally in the study plan and design. RAA collected the samples from the clinic, had carried out the clinical examination and ELISA laboratory work. RAA carried out the statistical analysis of data and reported the results of clinical examination and ELISA. WKBK and AGA collaborated on molecular work and in writing, revising, and improvement of the article for publication. All authors read and approved the final manuscript.
